# Spatiotemporal analysis of the dengue outbreak in Guangdong Province, China

**DOI:** 10.1186/s12879-019-4015-2

**Published:** 2019-06-04

**Authors:** Guanghu Zhu, Jianpeng Xiao, Tao Liu, Bing Zhang, Yuantao Hao, Wenjun Ma

**Affiliations:** 1 School of Mathematics and Computing Science, Guilin University of Electronic Technology, Guilin, 541004 China; 2 Guangdong Provincial Institution of Public Health, Guangdong Provincial Center for Disease Control and Prevention, Guangzhou, China; 3 Department of Medical Statistics and Epidemiology, School of Public Health, Sun Yat-sen University, Guangzhou, 510080 China

**Keywords:** Dengue, Cluster, Spatiotemporal transmission

## Abstract

**Background:**

Dengue is becoming a major public health concern in Guangdong (GD) Province of China. The problem was highlighted in 2014 by an unprecedented explosive outbreak, where the number of cases was larger than the total cases in previous 30 years. The present study aimed to clarify the spatial and temporal patterns of this dengue outbreak.

**Methods:**

Based on the district/county-level epidemiological, demographic and geographic data, we first used Moran’s I statistics and Spatial scan method to uncover spatial autocorrelation and clustering of dengue incidence, and then estimated the spatial distributions of mosquito ovitrap index (MOI) by using inverse distance weighting. We finally employed a multivariate time series model to quantitatively decompose dengue cases into endemic, autoregressive and spatiotemporal components.

**Results:**

The results indicated that dengue incidence was highly spatial-autocorrelated with the inclination of clustering and nonuniformity. About 12 dengue clusters were discovered around Guangzhou and Foshan with significant differences by district/county, where the most likely cluster with the largest relative risk located in central Guangzhou in October. Three significant high-MOI areas were observed around Shaoguan, Qingyuan, Shanwei and Guangzhou. It was further found the districts in Guagnzhou and Foshan were prone to local autoregressive transmission, and most region in southern and central GD exhibited higher endemic components. Moreover, nearly all of districts/counties (especially the urban area) have cases that were infected in adjacent regions.

**Conclusions:**

The study can help to clarify the heterogeneity and the associations of dengue transmission in space and time, and thus provide useful information for public health authorities to plan dengue control strategies.

## Background

Dengue is a mosquito-borne viral infectious disease, which is transmitted from one person to another through the bite of the infected female *Aedes aegypti* and *Aedes albopictus* [1]. In recent decades, dengue incidence has grown dramatically around the world [1], leading to half the world’s population being at risk of infection and 390 million people being infected each year [2]. It is now regarded as a big international public health concern. In China, dengue epidemics also exhibited clear upward trend both in extent and severity since its reemergence in 1978 in Guangdong (GD) Province, which has always been the hardest-hit Chinese province [3]. Particularly in 2014, an explosive outbreak of dengue unexpectedly attacked GD, in which the reported cases were more than 3 times of the total number in previous 20 years [4]. During this outbreak, it was observed that dengue is heterogeneously distributed in time and space, and the incidence varied greatly among districts and counties [4]. Characterizing the spatial-temporal heterogeneity of this dengue outbreak is very important, which allows to recognize the dengue cluster and the risky area, and further to improve dengue control and prevention strategies.

Many previous studies were conducted to discover and predict the spatial and temporal patterns of dengue by using mapping techniques [5–8], dynamical models [9, 10] or statistical methods [6, 7, 11, 12]. The recent dengue outbreak in China also drew similar analysis, such as inferring the spatiotemporal patterns of dengue transmission [6, 7, 9–11], and identifying the determinants of spatial variations in the dengue epidemic [6, 11, 12]. However, existing studies usually focused on one or several cities, and the spatiotemporal heterogeneity of the transmission patterns remains little understood at a finer scale of Guangdong.

This study addressed this issue by examining the spatiotemporal process at district/county level that contributed to the 2014 dengue outbreak in GD Province. We first used global Moran’ I to investigate the spatial autocorrelation of dengue incidence around the 128 districts and counties, and then employed Anselin Local Moran’s I (local indicators of spatial association [LISA]) and Kulldorff’s spatial scan statistics to detect the dengue clusters and identify the disease dynamics dispersion in these regions. Finally, we decomposed dengue cases into endemic, autoregressive and spatiotemporal components by a multivariate time-series model. The autoregressive and spatiotemporal components described an autoregression on past counts in the same and in other districts, respectively, which can capture occasional outbreaks and dependencies across regions [13, 14]. The endemic component captures the background risk of new events by external factors (independent of the history of the epidemic) [13, 14]. The results can help to clarify the spatiotemporal patterns of dengue transmission at 128 districts/counties of GD, which might assist in the development of dengue control and prevention strategies in this province.

## Methods

### Study site

**Fig. 1:**
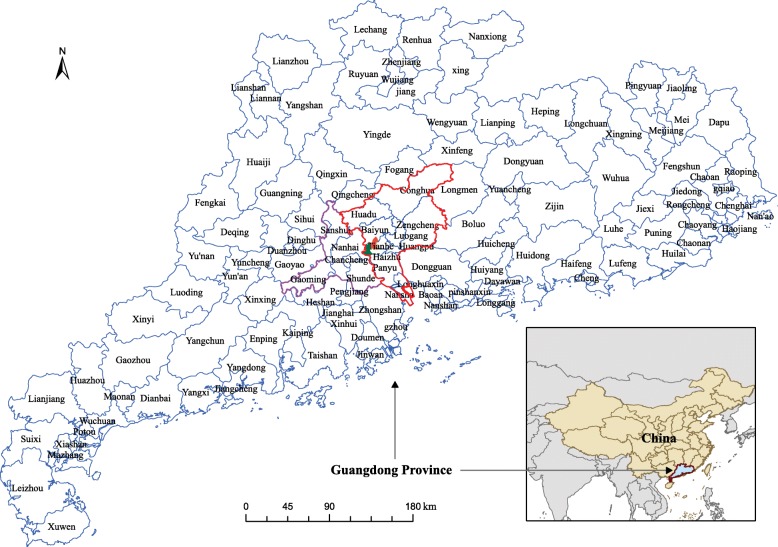
Location of the study area. The districts with blue and red colors are Yuexiu and Liwan, respectively. The regions with red and purple boundary are the cities of Guangzhou (the provincial capital of Guangdong) and Foshan, which accounted for 82.8% and 7.8% of the total dengue cases in 2014, respectively. The map is our own

GD Province was selected as the study area because it was the most seriously affected region by dengue in China. This province is situated in the southern China, which is an economic, finance, industry and transport center in China. It has an area of 179,800 km^2^ and about 100.7 million inhabitants. GD is administratively divided into 21 prefecture-level cities (see Fig. 5), including 128 counties/districts (see Fig. 1). The climate is subtropical humid, with short, mild, dry winters and long, hot, wet summers. The annual mean temperature is 21.8^∘^C and the annual accumulate precipitation is 1789 mm.

### Data collection

Dengue is a legally notifiable communicable disease in China since 1989. Dengue cases were diagnosed according to the unified diagnosis criteria issued by the Chinese Ministry of Health, including clinically diagnosed and laboratory confirmed cases. Dengue cases data reported between 2014 and 2018 was used in this study. The data was obtained from Guangdong Provincial Center for Disease Control and Prevention (GDCDC), with regard to the number of reported apparent and confirmed dengue cases per county/district. The MOI data is retrieved from GDCDC. In GD, mosquito ovitraps are placed widely to monitor mosquito density. MOI is computed as the proportions of positive mosquito ovitraps. Population data for each county/district in GD in 2014 were retrieved from the Guangdong Statistical Yearbook.

### Data analysis

#### Spatial autocorrelation analysis

The spatial autocorrelation of the dengue numbers was evaluated by using Global Moran’s I statistic, which can measure the correlation among spatial observations, and allows to find the global pattern (clustered, dispersed, random) among regions. The formula is defined as [15]

$$I=\frac{n\sum\nolimits_{i,j}W_{ij}\left(x_{i}-\overline{x}\right)\left(x_{j}-\overline{x}\right)}{\sum\nolimits_{i,j}W_{ij}\sum\nolimits_{i}(x_{i}-\overline{x})^{2}}, $$

where *n* is the number of spatial units indexed by *i* and *j*; *x*_*i*_ is the variable of interest in spatial unit *i*; $\overline {x}$ is the mean of *x*; *w*_*ij*_ is the spatial weight of relationship between units *i* and *j*. The above formula can provide an index of dispersion from -1 to +1, corresponding to maximum negative and positive autocorrelation, respectively [15].

#### Dengue clustering

The presence of dengue clustering was identified by two cluster detection programs: Anselin’s Local Moran I (LISA) test statistics and Kulldorff’s spatial scan method. Using the input area data (case, population and geographic information), they can output the location, approximate size and significance level of identified clusters. Based on the spatial time series of reported cases, the evolution is divided into in four periods (from June to August, September, October, and from November to December).

First, LISA was used to identify the localized clustering with specific districts. For the spatial unit *i*, LISA is computed as [16]

$$I_{i}=\frac{x_{i}-\overline{x}}{S^{2}}\sum\limits_{j}W_{ij}\left(x_{j}-\overline{x}\right), $$

where *S*^2^ is the variance of *x*. A positive or negative value of *I*_*i*_ means spatial clustering of similar or dissimilar incidence rate, which allows to classify regions into five categories: high-high, low-low, high-low, low-high, and non-significant. The high-high and low-low areas indicated the hot and cold spots of dengue incidence, respectively. While the high-low and low-high areas were the outliers [16].

Next, Kulldorff’s spatial scan statistics were used to identify the specific clusters of varying sizes within the study area, which was implemented by SaTScan v9.4.4 (https://www.satscan.org/). The statistic software can generate circles consisting of regions whose incidence rates are significantly higher than the regions outside the circles [17]. This was achieved by gradually scanning a window across space, and the window with the maximum likelihood was the most likely cluster. A Poisson-based model was used for this study. This method has previously been validated for plotting and understanding local spatiotemporal clusters of many epidemics, such as dengue [18, 19] and malaria [20]. Here the maximum cluster size was set to 5% of the total population at risk.

#### Endemic-epidemic multivariate time-series model

The endemic-epidemic multivariate time-series model proposed by Held and Paul [13, 14], is designed for analyzing the spatiotemporal components of surveillance data. Let *Y*_*it*_ denote the number of cases in region *i* at time *t*, which is assumed to follow negative binomial distribution with conditional mean

1 $$  u_{it}=e_{i}\nu_{it}+\lambda_{i}Y_{i,t-1}+\phi_{i}\sum\limits_{j\neq i} \omega_{ji}Y_{j,t-1},  $$

and overdispersion parameter *ψ*_*i*_>0 such that the conditional variance of *Y*_*it*_ is *μ*_*it*_(1+*ψ*_*i*_*μ*_*it*_). In Eq. (1), *e*_*it*_*ν*_*it*_ is the endemic component, which is used to models seasonal variation and trends; the other two components are the observation-driven epidemic components: an autoregressive component *λ*_*it*_*Y*_*i,t*−1_ at previous time step (reproduction of the disease within unit *i*,), and a spatiotemporal component $\phi _{it}\sum \nolimits _{j\neq i} \omega _{ji}Y_{j,t-1}$ (transmission from other units) [14]. The three components have log-linear predictors of the forms:

$$\log(\nu_{it})=\alpha^{(\nu)} + b_{i}^{(\nu)}+\sum\limits_{s}[\gamma_{s}\sin(\omega_{s}t)+\delta_{s}\cos(\omega_{s}t)], $$

$$\log(\lambda_{i})=\alpha^{(\lambda)} + b_{i}^{(\lambda)}, $$

$$\log(\phi_{i})=\alpha^{(\phi)} + b_{i}^{(\phi)}, $$

where *α*^(*x*)^ is intercept, and $b_{i}^{(x)}$ (*x*=*ν*,*λ*,*ϕ*) is regional random effect which accounts for geographic heterogeneity. The endemic log-linear predictor *ν*_*it*_ incorporates a periodic wave of frequency by letting *ω*_*s*_=2*π*/52 for weekly data in this paper. The spatiotemporal weight *ω*_*ji*_ which describes the transmission strength from unit *j* to *i* is assumed to follow a well-recognized power-law distance decay [11, 21]. The analysis was carried out by the “hhh4” model provided in R package surveillance [13, 14].

## Results

### Descriptive analysis

**Fig. 2:**
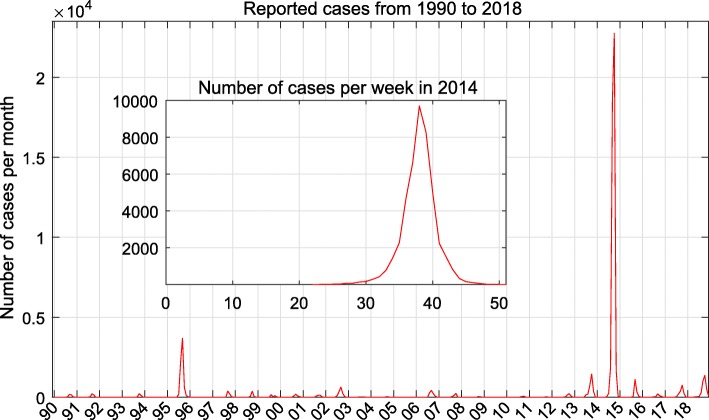
Monthly number of dengue cases reported in Guangdong Province from 1990 to 2018. The inside figure shows the weekly number of dengue cases in 2014

**Fig. 3:**
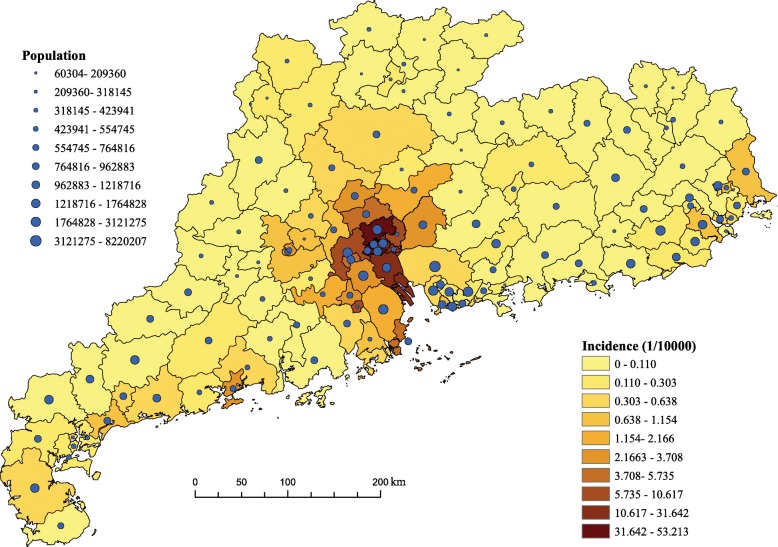
The geographical distribution of dengue incidence and population size at county/district level in Guangdong Province in 2014. The map is our own

As shown in Fig. 2, during 1990 and 2013, about 16 thousand indigenous dengue cases were recorded in GD Province. In 2014, the number hit a historic record, where 45,123 cases were reported in GD, accounting for 0.042% of the total population. Since then, dengue incidence stayed in a relatively high level, as 8,281 cases were notified in GD during 2015 and 2018. In each year, cases occurred like convex curve through Summer and Autumn. For the big outbreak in 2014, the indigenous cases were first reported in mid-June, with slow increase in July and August. The number in these three months was 1942 cases. A rapid increase and the peak of the epidemic were observed in September and October, which accounted for 17,405 and 23,905 cases, respectively. During these two months, the dengue infections displayed clear spatial expansion trend. After that, the dengue incidence reduced significantly, where the number reported in November and December was 1871 cases. At district/county level, the largest number of cases occurred in Baiyun district, followed by Haizhu, Yuexiu and Liwan districts, all of which located in the city of Guangzhou. The Geographical distributions of dengue cases were shown in Fig. 3.

### Spatial autocorrelation

Table 1 shows a significant positive spatial autocorrelation of dengue incidence for the four periods, where the values of global Moran’s I ranged from 0.164 to 4.999. It means that the districts/counties close together tend to have similar baseline of incidence rates, and clustered patterns existed during this outbreak. Such phenomenon was more evident in the highly epidemic period, i.e., September and October.

**Table 1:** Spatial autocorrelation statistics on dengue epidemics in Guangdong (GD) Province in 2014

Period	Incidence(1/10000)	Moran’s I	z-score	*p*-value
June-August	0.184	0.164	3.327	< 0.01
September	1.651	0.499	8.375	< 0.01
October	2.267	0.494	8.517	< 0.01
November-December	0.177	0.369	6.778	< 0.01

### Spatial and temporal clustering

**Fig. 4:**
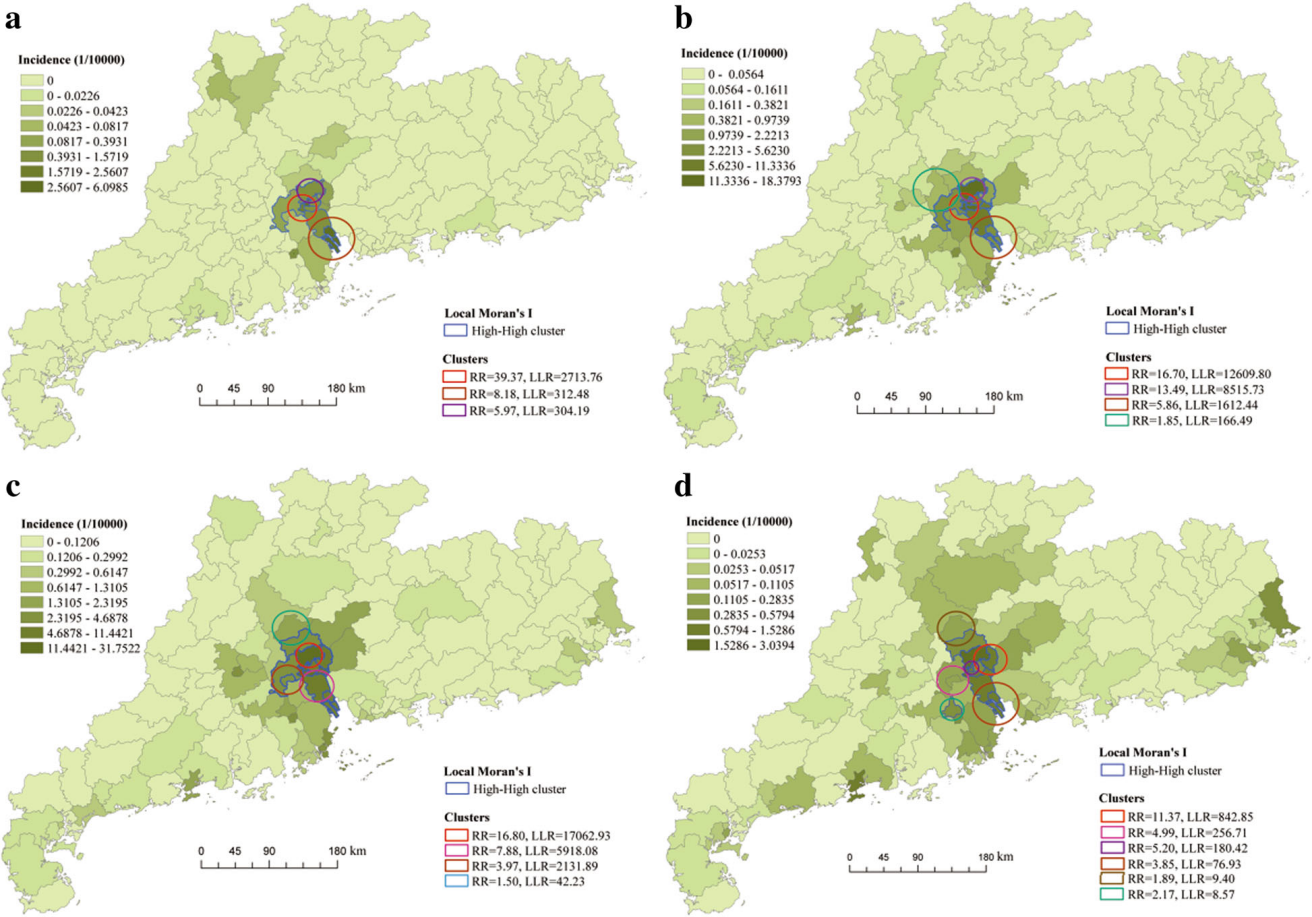
SaTScan-generated cluster circles and local Moran’s I clusters overlain on the map of dengue incidence in Guangdong Province for the study period: (**a**) June - August, (**b**) September, (**c**) October, and (**d**) November - December of 2014. The RR and LLR represent relative risk and log likelihood ratio, respectively. The maps are our own

Figure 4 shows the SaTScan-generated cluster circles and local Moran’s I clusters overlain on the map of dengue incidence in GD Province in the four periods. Based on the LISA statistics, it is observed that the high-high clusters only exhibited in the districts/counties of Guangzhou and Foshan, suggesting that high incidence rates potentially occurred these regions with interactive transmission. During June and August, the high-high clusters first emerged in the 8 districts of Guangzhou (Yuexiu, Liwan, Haizhu, Baiyun, Tianhe, Huangpu, Panyu, and Nansha). As time went by, the clusters geographically expanded to the west and the north. The cluster covered another two districts (Luogang and Nanhai) in September, as well as Huadu and Chancheng in October. During November and December, in addition to adding a new district (Pengjiang), the clusters restricted in Guangzhou (except Zengcheng and Conghua).

**Fig. 5:**
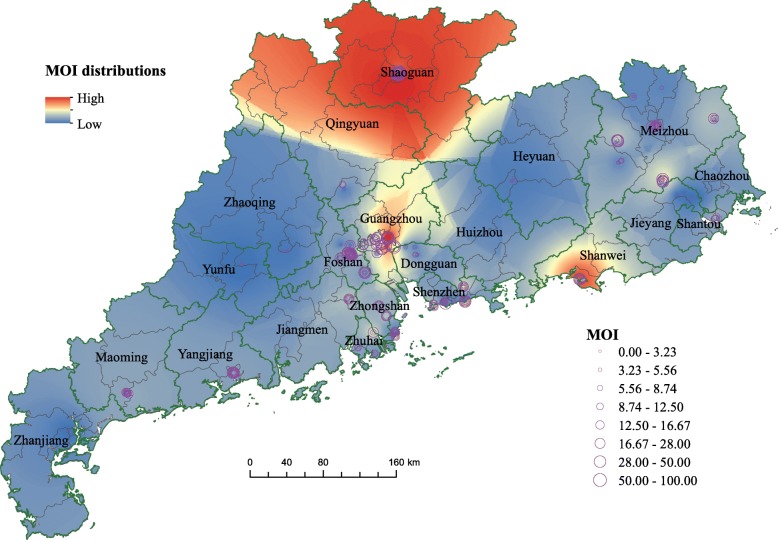
The marked oviposition positive index (MOI) data during July and October 2014 in Guangdong and the corresponding estimation of the spatial MOI distributions by using inverse distance weighting. The labeled name is the 21 prefecture-level cities. The map is our own

Based on the retrospective scan analysis by SaTScan, many clusters with statistically significant high incidence in different periods were detected. As shown in Fig. 4, during June and August, three significant clusters were detected, which covered 7 districts (Liwan, Yuexiu, Haizhu, Baiyun, Nansha, Panyu and Tianhe). In September, another new cluster was observed in Foshan (Nanhai, Sanshui and Sihui). In October, the clusters cover more districts in Foshan and Guangzhou (Huangpu, Chancheng, Nanhai, Qingcheng and Huadu), as well as Qingcheng District in Qingyuan. After that, the clusters scattered around cities of Guangzhou, Foshan, Qingyuan and Jiangmen, will relatively low relative risk and small log likelihood ratio. The first likely cluster existed in October, as a circle center in Baiyun district and a radius of 16.03 km to include Yuexiu and Tianhe districts, with 10,648 observed cases (1090.96 expected). The second likely cluster existed in September, as a circle center in Liwan district and a radius of 17.41 km to include Yuexiu, Haizhu and Tianhe districts, with 7,943 observed cases (832.89 expected). It was observed that the most likely clusters through the four periods were always concentrated in the city of Guangzhou, but as the infection propagated, the cluster center vary from Liwan, Baiyun, to Luogang, with different radius and relative risk.

### Spatial MOI distributions

Figure 5 illustrate the spatial distributions of MOI. About 6 hundred records of MOI are collected during July and October 2014. These records were marked around GD, mostly in Guangzhou, Foshan, Shenzhen, Zhongshan, Zhuhai and Dongguan), which are also the high dengue prevalence areas in recent decade. Based on these records, it was found three clusters with high MOI. locating in Shaoguan and Qingyuan, Shanwei, and Guangzhou, respectively.

### Endemic-epidemic components

**Fig. 6:**
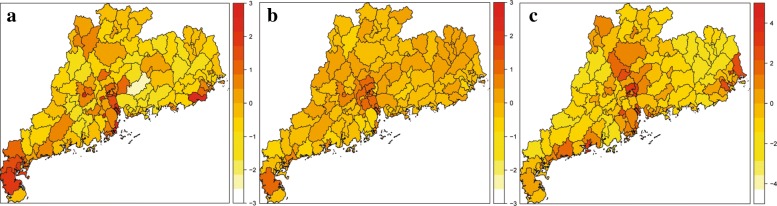
Maps of the estimated random intercepts at district/county level based on a multivariate time series model, which is divided into three parts: (**a**) spatiotemporal, (**b**) autoregressive and (**c**) endemic components. The maps are our own

**Fig. 7:**
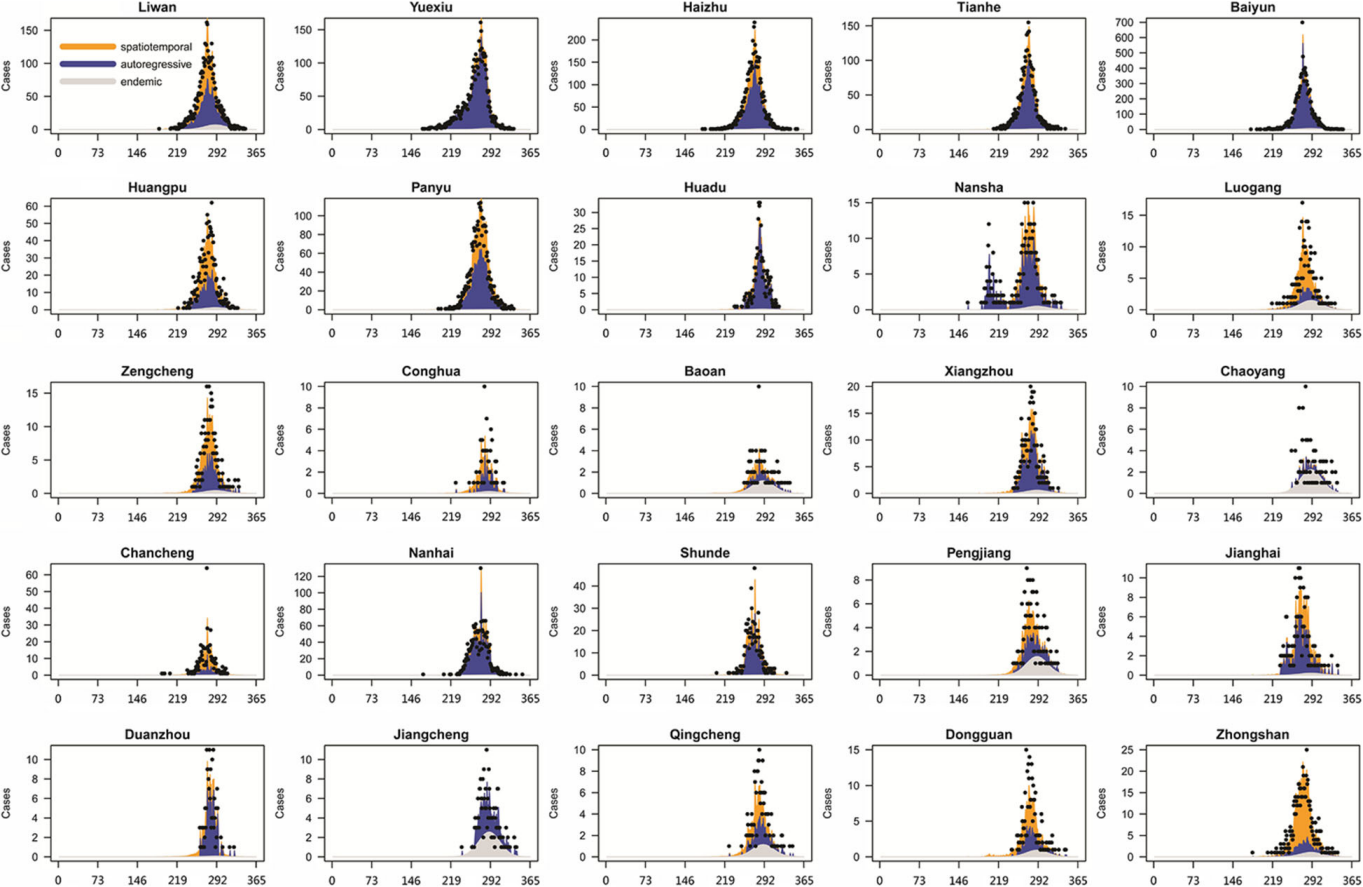
Fitted components of dengue cases in 25 districts/counties with most cases by a multivariate time series model. Black dots are drawn for weekly counts. The light gray, blue, and orange area shows the estimated endemic, autoregressive, and spatiotemporal contributions. X-axis represents the days in the whole year 2014

Figures 6 and 7 show that the endemic-epidemic components of dengue data by the multivariate time series model, where the cases were decomposed into endemic, autoregressive and spatiotemporal components.

The variances of the random effect for the three parts were estimated to be 0.485, 1.731 and 2.635, respectively. It means that there was little variation in the autoregressive component, but vast variation in the endemic and spatiotemporal components. As shown in Fig. 6, it is found a homogeneously low random effect of the autoregression in most of the districts, but a little high value in Guagnzhou and Foshan, as well as Leizhou district, indicating that these regions were affected by large local outbreaks. Further, it is observed that the random intercepts of the endemic and spatiotemporal components exhibited significant heterogeneity across districts, in which southern and central GD admitted a relatively high endemic incidence with a few cases from other districts.

Figure 7 shows the time series of fitted components for the 25 districts/counties with most dengue cases. Some special features are observed: (1) Dengue cases in Yuexiu, Baiyun, Huadu and Nanhai have vast autoregressive components, indicating that these districts were predominantly affected by the previous infection in their own regions; (2) Baoan, Chaoyang and Jiangcheng have extremely big endemic distributions and thus admitted high risk of local transmission; (3) Dengue cases in most districts have spatiotemporal components of dengue cases, especially in Huangpu, Luogang, Zengcheng, Dongguan and Zhongshan, indicating that these districts easily suffered dengue infection from the adjacent districts; (4) Liwan, Haizhu, Tianhe, Panyu, Nansha, Zengcheng, Conghua, Xiangzhou, Chancheng, Shunde, Pangjiang, Jianghai and Qingcheng have similarly big components of autoregressive and spatiotemporal incidence, which means that the risk of dengue infection in these districts mainly come from local and neighboring regions.

## Discussion

This study explored the spatiotemporal variation and association of the dengue transmission in GD Province in 2014. By using Moran’s I statistics and Spatial scan method, as well as a multivariate time series model, we were able to clarify the spatiotemporal distribution patterns, detect the dengue clusters, and evaluate the spatiotemporal components of dengue infections. To our knowledge, this is the first attempt to infer the dengue transmission patterns in GD Province at district/county level. Several notable findings could provide meaningful clues for public health authorities to implement effective interventions on dengue infection.

We found a significant positive spatial autocorrelation on the dengue incidence in the four time periods. Further analysis on the spatial autocorrelation of the yearly incidence between 2015 and 2018, yields the global Moran’s I as 0.147, 0.041, 0.139 and 0.315, respectively. These positive values highlight that the districts and counties at geographical proximity shared a similar level of vulnerability to dengue, and such feature was more evident as the disease developed. In other words, dengue infections do not spread uniformly or randomly but occurred in cluster, and thus suggest possible measures of early detection for disease surveillance. Our results are consistent with previous findings that dengue is inclined to spatially correlate with clusters [8]. It is possible because that dengue transmission are affected by a series of factors related to environment, climate, society, demography and vectors [6, 22, 23], and such factors have similar attributions in adjacent sites.

**Fig. 8:**
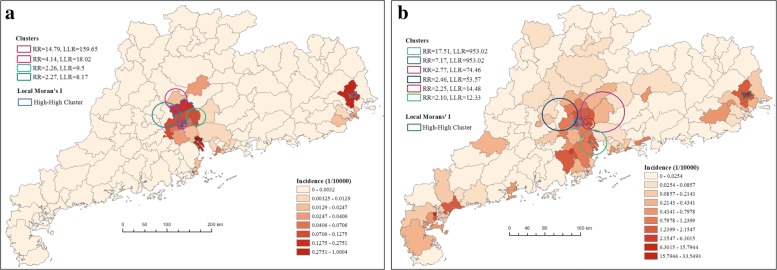
SaTScan-generated cluster circles and local Moran’s I clusters overlain on the map of dengue incidence in Guangdong Province in (**a**) 2016 and (**b**) 2017. The RR and LLR represent relative risk and log likelihood ratio, respectively. The maps are our own

To locate the hotspots for future surveillance strategy, we identified the prominent spatial clustering covering specific districts. In 2014, though dengue infection rapidly spread over most of districts in GD, it was highly localized in particular locations and times. We identified 12 significant clusters during the four periods, which included about 22 districts lied in the cities of Guangzhou, Foshan, Qingyuan and Jiangmen. The most likely cluster with the largest relative risk occurred in central Guangzhou in October. Both cluster detection methods identified similar significant clustering, suggesting that the result is robust. It is found that the cluster center, the radius, and the relative risk varied across different districts as the infection propagated, but the 5 districts, i.e., Liwan, Yuexiu, Haizhu, Tianhe and Baiyun, always recorded in clustering, indicating their important role in dengue transmission. Compared to other regions, eastern Foshan, southwestern and central Guangzhou are more vulnerable to dengue, which appeared as a dengue hotspot in mapping and cluster assessment. The outbreak information in one district of the hotspots can serve as an early warning device for a possible outbreak in neighboring districts. Our findings are consistent with previous analysis, where they found similar dengue clusters at rough scale [7, 9–12]. To further verify the location of dengue clusters, we performed similar analysis on the incidence data in 2016 and 2017. As shown in Fig. 8, beside a new cluster observed in east GD (Chaoan and Xiangqiao), the districts in Guangzhou (except Nansha, Panyu and Conghua) and in Foshan (except Gaoming) are also marked as the hot regions. The almost overlapped clusters in recent years indicated that the marked hotspots are not made by chance. Possible reasons that contribute to such clustering patterns could be: (1) Southwestern and central Guangzhou is the downtown, with particular features: dense population density [4], higher temperature (due to the urban heat island effect) [10], in the process of urbanization [6], and a transportation hub [4, 10]; (2) Eastern Foshan is near Guangzhou, which share large human mobility between these two regions [9, 12].

Using the recorded MOI data, we found three significant high-MOI areas located in Shaoguan, Qingyuan, Shanwei and Guangzhou. The proportions of positive mosquito ovitraps in Shaoguan and Qingyuan are almost ninety percent, which signifies high mosquito density there. However in reality, these regions admitted low dengue incidence. That is probably due to the sparsity of human population and human movement, yielding seldom circulation of dengue viruses. But in Guagnzhou, relative high MOI combined with the above-mentioned features easily triggers dengue occurrence.

By fitting a multivariate time series model to dengue cases, we further clarified the spatiotemporal pattern of dengue transmission. First, we found significant spatial variation in the endemic and spatiotemporal component across the districts, while the autoregressive component was more spatially homogenous, similarly to the patterns in city level [11]. Second, we observed that the autoregressive, endemic and spatiotemporal components have different contributions to each district/county. It is important to note that nearly all districts have cases that were infected in adjacent regions. Such feature is more evident in and around urban area, possibly because frequent human mobility resulted in many interconnected infections between these places. The result reminds us that joint control efforts in surrounded regions could be necessary for dengue intervention. As a matter of fact, simultaneous intervention in multi-area since October 2014 had significant effect to control the dengue outbreak in Guangzhou [24]. For those districts with more autoregressive components (Yuexiu, Baiyun, Huadu and Nanhai), rigorous intervention strategies should be implemented in case of sudden local infection. For those districts with more endemic component, active surveillance should be adopted since they are more susceptible to dengue infection.

The spatiotemporal analysis presented in this paper differs from the existing studies, both in methodology and scope. By using Richards model and G* statistic [7], wavelet analysis [9], and compartmental model [10], these researchers found that the initial hot spot for the outbreak was Yuexiu district, and then the disease spread to its neighboring districts in Guangzhou and other cities in GD province in 2014. By using Moran’s I method and geographical detector, Cao et al. [6] found that central Guangzhou was the hot spot of dengue infection, and that temperature, precipitation and road density were the main factors leading to dengue transmission in Guangzhou in 2014. By using geographically weighted regression model, Ren et al. [12] found that population size, road density, and economic status are the determinants of spatial variability of the 2014 dengue epidemic across Guangzhou and Foshan. Cheng et al. [11] decomposed dengue risk in the 21 cities of GD by a multivariate time series model. They found that endemic component contributed much more in the Pearl River Delta area, while areas with relatively low incidence are highly dependent on spatiotemporal and local autoregressive spread. These analyses were mainly based on the city level of GD [6, 7, 9, 11] or district level of Guangzhou [7, 10, 11]. Our study focus on the 128 districts and counties of GD, a finer scale and a larger area, allowing for better understanding dengue transmission patterns.

Several limitations existed in our study worth noting. (1) The incidence data are based on passive surveillance system. We were unable to calculate the unreported and asymptomatic cases. (2) The cases were recorded in the residence of the patients, which could lead to inconformity with the real location where they were infected. (3) The statistic model employed here could not explicitly incorporate climatic and socio-ecological factors, such as climate and human migration. More efforts are needed to assess the effects of these factors on the spatiotemporal evolutions of dengue.

## Conclusions

This paper analyzed the spatiotemporal diffusion patterns contributed to the large dengue outbreak in GD in 2014. The results showed that dengue distribution was strongly correlated in space, and was highly clustered around Guangzhou and Foshan, with a slight movement of clustering over time. Further analysis revealed that autoregressive, endemic and spatiotemporal transmission in each district and county had different contributions to human infection, and most of districts/counties (especially the urban area) had cases that were infected in adjacent regions. This study can help to clarify the heterogeneity and the associations of dengue transmission in time and space, and thus provide insightful information for public health authorities to plan dengue control strategies.

## Abbreviations

GD: Guangdong
GDCDC: Guangdong Provincial Center for Disease Control and Prevention
LISA: Local indicators of spatial association
MOI: Mosquito ovitrap index
